# Good Pay for Good Men

**Published:** 1911-03

**Authors:** 


### Good Pay for Good Men

THE health department of Buffalo has sent to the board of aldermen a communication calling attention to the pressing need of enlarging the commissioner's staff of assistants and the necessity for a general increase of salaries in the department.

The proposed advances are:

Commissioner, $4,000 to $5,000; assistant, $2,000 to $3,000; secretary, $1,800 to $2,000; chief clerk, $1,300 to $1,500; registrar, $1,500 to $1,700; chief inspector of food and drugs, $1,400 to $1,700; chief sanitary inspector, $1,400 to $1,700; bacteriologist, $2,000 to $2,200; assistant bacteriologist, $1,700 to $2,000. Raises are also asked for assistants, clerks and others.

The additional help asked are: Four additional food and drug inspectors at $1,000 each; three medical sanitary inspectors, $1,000 each; an examiner in lunacy, at $1,000, to comply with the new law requiring the health department to examine all insane or suspected insane persons before they are committed; two school dental examiners (after September 1), at $1,000 each: eight more school nurses at $600 each; six nurses, as requested by the District Nurses' Association, at $720 each; two clerks, at $900 each; two stenographers, at $900 each.

Some of the additions to the staff are made necessary by recent changes in the state laws which are practically compulsory. Many of the increases in salary were asked for by Dr. Ernest Wende when he was health commissioner and were held up by the aldermen from purely party motives at a time when political red-fire was being burned at the shrine of economy in an effort to keep down that elusive and fanciful fetish known as the tax rate. The increases now asked for will amount to about $30,000 per year which in itself and likewise to a man who cannot bring himself to think outside the realm of the common copper cent, seems like a lot of money; but which when applied to the health needs of a city of close on to half a million human beings, is a remarkably modest sum.

It requires little effort to hark back to those golden days of budding health department efficiency when a city bacteriologist was asked at the hands of the aldermen and recall the violent opposition of one official in particular to the employment of what he facetiously chose to call a "bug chaser" during those brief intervals which lie abreast his dollar-hunting moments when he gave some contracted thought to the more serious duties of his aldermanic life. Nor does it require effort to recall the change wrought in this same alderman a few weeks later when he became a devoté at the feet of science and begged his fellows to give the health department what it asked for. It wasn't in a broad spirit of voluntary humanity to the many that he begged this boon. It was selfishness because he had seen a child led to the brink of the grave—a child choking in the grip of diphtheria and because a wise physician had taken the gift of opportunity at the hands of Providence and shown him the danger of delay in diagnosis. It required just such an object lesson to pierce the density of ignorance which clad this chested ward patriot, and it served to provide a city bacteriologist.

Buffalo is in fairly creditable condition healthwise at the present time; but it is a rapidly growing city and the need of careful and constant supervision of its health is pressing. It were easy to live Chinese fashion and lag along leaden-legged at the heel of destiny, but it is criminal in its negligence. There should be no hesitation in granting the increases in pay requested, for the present salary list is wretchedly inadequate; nor should there be any question regarding the necessity of increase in the numerical strength of the staff. The health of Buffalo demands a department of the highest efficiency and mobility, and this cannot be achieved by paying miserly salaries to able men or pursuing a pinchbeck policy in economy of numbers.

